# Evaluation of Selected Immunomodulatory Glycoproteins as an Adjunct to Cancer Immunotherapy

**DOI:** 10.1371/journal.pone.0146881

**Published:** 2016-01-22

**Authors:** Bhagwant Kaur Sekhon, Rebecca Heidi Roubin, Yiming Li, Parimala B. Devi, Srinivas Nammi, Kei Fan, Daniel Man-yuen Sze

**Affiliations:** 1 Faculty of Pharmacy, The University of Sydney, New South Wales, 2006, Australia; 2 School of Medical Sciences and Health Innovations Research Institute (HIRi), RMIT University, Victoria, 3000, Australia; 3 School of Medical Science, University of Western Sydney, Campbelltown, New South Wales, 2560, Australia; University of Strathclyde, UNITED KINGDOM

## Abstract

Polysaccharopeptide (PSP), from *Coriolus versicolor*, has been used widely as an adjuvant to chemotherapy with demonstrated anti-tumor and broad immunomodulating effects. While PSP’s mechanism of action still remains unknown, its enhanced immunomodulatory potential with acacia gum is of great interest. Acacia gum, which also contains polysaccharides and glycoproteins, has been demonstrated to be immunopotentiating. To elucidate whether PSP directly activates T-cell-dependent B-cell responses *in vivo*, we used a well-established hapten carrier system (Nitrophenyl-chicken gamma globulin (NP-CGG)). 6-week C57BL/6 male mice were immunised with 50 μg of NP_25_-CGG alum precipitate intraperitoneally. Mice were gavaged daily with 50mg/kg PSP in a vehicle containing acacia gum and sacrificed at days 0, 4, 7, 10, 14 and 21. ELISA was used to measure the total and relative hapten-specific anti-NP IgA, IgM and IgG titre levels compared to the controls. It was found that PSP, combined with acacia gum, significantly increased total IgG titre levels at day 4 (P< 0.05), decreased IgM titre levels at days 4 and 21 (P< 0.05) with no alterations observed in the IgA or IgE titre levels at any of the time points measured. Our results suggest that while PSP combined with acacia gum appears to exert weak immunological effects through specific T-cell dependent B-cell responses, they are likely to be broad and non-specific which supports the current literature on PSP. We report for the first time the application of a well-established hapten-carrier system that can be used to characterise and delineate specific T-cell dependent B-cell responses of potential immunomodulatory glycoprotein-based herbal medicines combinations *in vivo*.

## Introduction

Evading immune destruction has been deemed as an emerging hallmark of cancer [1]. In particular, the absence of strong cancer antigens, loss of MHC class I, or stimulatory and or co-stimulatory molecules can result in the failure of T-cell recognition, antigen presentation and tumor elimination [2,3]. The side-effects associated with chemotherapy places additional burden on the host’s ability to mount an effective immune response against antigens and the compromised immune system is therefore vulnerable to potentially life-threatening infections [4]. Attempts at targeting and stimulating the suppressed host’s immune response against such assaults has been met with mixed success in the delivery of effective therapies. Although various immunotherapeutic approaches such as dendritic cell vaccination possess low toxicity, unfortunately they rarely exceed an objective clinical tumor response rate of 15% [5]. Therapies such as tumor-infiltrating lymphocytes (TILs) protocols have been recently evaluated in a pooled analysis reporting a 20% complete response rate with a 70% overall objective response rate in patients with melanoma [6]. Cytokine therapies, in particular the systemic administration of IL-12 have been associated with severe side effects and a very narrow therapeutic index [7]. Few such clinical trials administering IL-12 in cancer patients have showed encouraging results [7]. However, immunotherapy with other interleukins such as high dose IL-2 remains to be an effective treatment for the last 20 years in patients with metastatic renal cell carcinoma, conferring well-documented complete durable responses in 10–15% of select patients [8]. Antibody mediated therapies such as the development of monoclonal antibodies have been used to successfully target various receptors including VEGF, EGFR, Her-2 exerting various effects on different cancers resulting in prolonged survival [9]. In particular, the development of the fully humanized IgG1 monoclonal antibody ipilimumab targets the cytotoxic T lymphocyte antigen-4 (CTLA-4) on the surface of helper T-cells thereby inhibiting the development of immune tolerance. Ipilimumab is approved by the FDA for the treatment of melanoma [10]. More recently, the discovery of MDX-1106 (nivolumab), a fully humanised IgG4 checkpoint inhibitor targets programmed death 1(PD-1) exerting immunosuppressive effects on T-cells preventing tolerance. Phases I and II studies have been carried out on various cancers, showing objective tumor responses and improving overall survival of advanced melanoma patients [11]. As a result, nivolumab has been approved by the FDA for treating patients with unresectable or metastatic melanoma who are no longer responsive to other drugs, and patients with metastatic squamous non-small cell lung cancer (NSCLC) with progression on or after platinum-based chemotherapy. The aim is to develop and combine various therapeutic strategies together that deliver broad spectrum effects with potentially less toxicity while minimizing resistance, thereby improving treatment outcomes for those with cancer. Thalidomide is one such drug used for the effective management of multiple myeloma, possessing complex but multifaceted effects including potent cytotoxic and immunomodulatory activities [12]. In particular, thalidomide has the ability to stimulate prognostically significant cytotoxic T-cells responses in patients with multiple myeloma [13] and Waldenström macroglobulinemia [14].

Numerous nutritional interventions have demonstrated potential in modulating the dsyregulated immune response. In particular, mushroom derivatives have been well studied and are known to possess immune-stimulatory effects, but clear evidence of their ability to augment immunological responses against specific antigens is slowly emerging [15]. Protein-bound polysaccharides or polysaccharopeptides (PSP), are a class of compounds found abundantly in certain mushrooms and have been widely used as biological response modifiers (BRM) in East Asia [16]. PSP derived from *Coriolus versicolor* has been reported to enhance the immune system, and be a potent immunomodulator against cancers and infections [17–19]. Immunostimulating effects of PSP include elevation of pro-inflammatory cytokines such as interferon γ (IFN-γ), interleukins such as IL-2; natural killer (NK) cell activity, activation of complement (C3), and T-cell proliferation [20]. Importantly, we previously reported that PSP upregulates the percentage of monocytes but did not alter T-cell subsets, NK cells or B-cells[21]. In particular, we did not find any significant differences in the CD4/8 ratio after PSP treatment [21]. This finding supports clinical studies that have reported no significant changes in the CD4/8 ratio after PSP treatment [22,23]. While our previous study on PSP was conducted in vitro, it did not take into account the issues of bioavailability or its effects on liver metabolism. It is plausible that the negative T-cell results may be attributable to the absence of a completely intact immune triad of DC, T and B-cells. A confirmatory study using animals is therefore crucial and the subject of this study.

**Fig 1:**
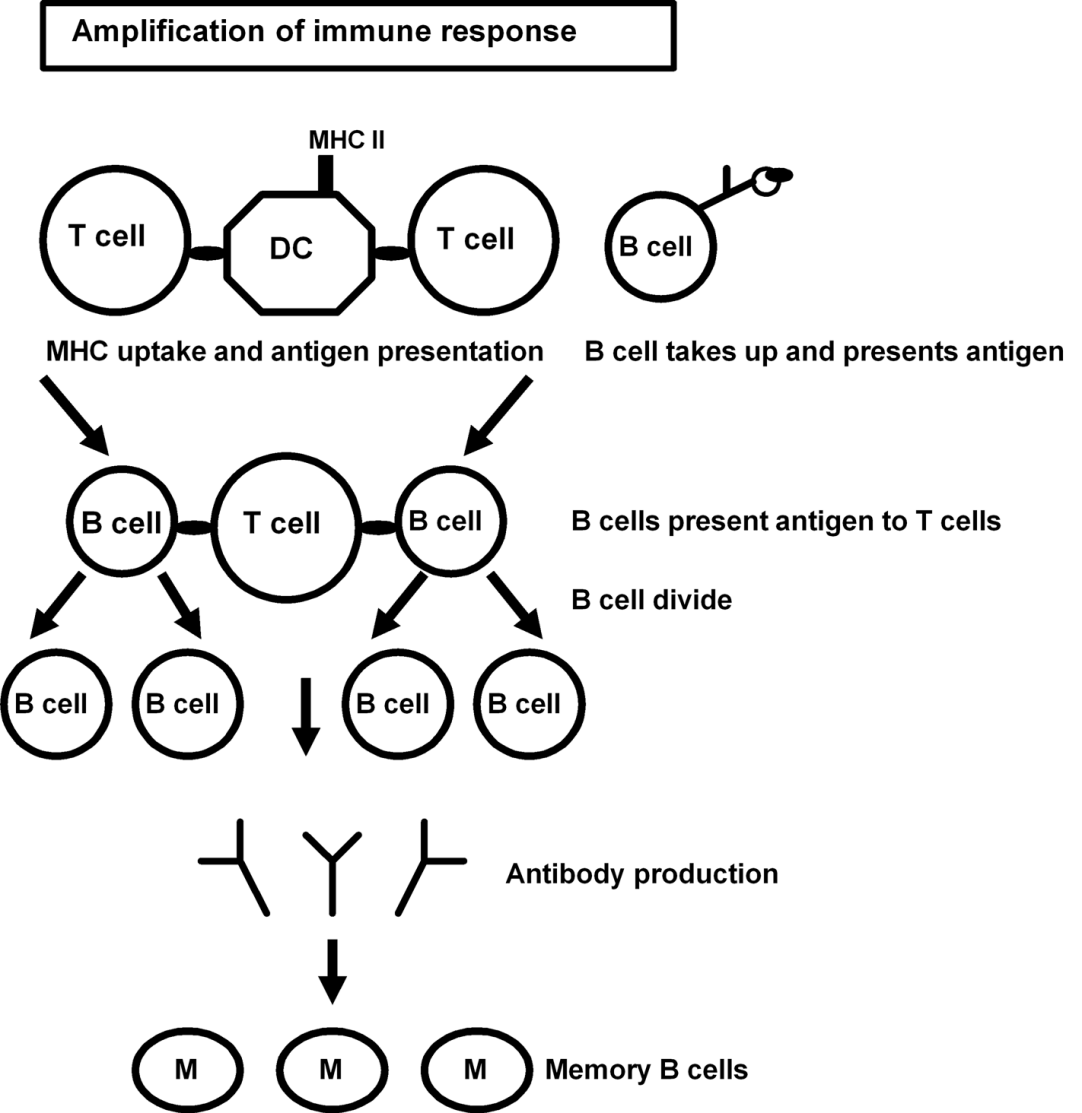
Schematic diagram of the events that give rise to cognate interactions between T-cell and B-cells.

In T-cell dependent B-cell antibody responses, an antigen taken up by DC or macrophages are presented to naïve T-helper cells where cognate interaction between B-cells can occur. The B-cell with the same specificity as a particular T-cell engulfs and digests the antigen and displays the antigenic fragments bound to its unique MHC II molecules on its surface for further presentation. A mature matching T-cell interacts with the B-cell and secretes cytokines leading to cell division of B-cells and antibody production. This process may lead to the production of memory B-cells (Fig 1).

Interestingly, much of the data (including our own) suggest an augmentation of cytokine levels and immunological subsets conferred by PSP’s non-specific but broad ranging activities. Whether PSP exerts T-cell dependent B-cell antibody responses, or T-cell independent B-cell antibody responses has not been elucidated partly due to a lack of appropriate assays including animal models. An attempt to evaluate adjuvant effects of vaccination with glycoprotein-based herbal medicines with immunomodulatory potential (particularly *Astragalus* and PSPs from *Coriolus versicolor*) paired with KLH conjugates have been conducted [24,25]. While this study was able to demonstrate significant adjuvant effects with these herbal medicines, the KLH models present a number of limitations. Firstly the B-cell response to the complex KLH protein is a polyclonal response in relation to a wide repertoire of antigenic epitopes making the determination of specific responses such as T-cell dependent B-cell responses impossible. Secondly this T-cell dependent B-cell response in the KLH model spans an extended duration rendering it difficult to differentiate between direct and indirect effects. The adjuvant effects of vaccinating with glycoprotein-based herbal medicines in this KLH study are likely to be broad and non-specific. Therefore the NP-CGG model addresses both these limitations of the KLH model in being able to determine direct and specific T-cell dependent B-cell adjuvant effects of immunomodulatory herbal medicines combined with immunopotentiating agents. In order to maximise the splenic immune response, NP-CGG was administered via the i.p. route. As this is a mechanistic study to confirm adaptive immunity, there is no eventual clinical application for the NP-CGG model via the i.p. route itself. By measuring the specific anti-NP responses at different time points, a synchronous T-cell-dependent B-cell response can be outlined clearly.

We report here for the first time the application of a well-established hapten-carrier system NP-CGG to characterize and delineate the specific T-cell dependent B-cell immunological responses in the presence of PSP, a commonly used adjuvant to cancer immunotherapy. T-cell dependent B-cell antibody responses in particular have enormous potential for conferring adaptive immunity against cancer antigens (increased avidity) [26–28]. Therefore PSP’s ability to exert T-cell dependent responses *in vivo* is therefore highly desirable and has been investigated in this study.

We hypothesized that in the NP-CGG model, PSP exerts its immunological effects through T-cell dependent B-cell interactions by altering the subsequent immunoglobulin (Ig) isotype class as well the quantity of the Ig in animals initially challenged by NP-CGG and subsequently given PSP.

While it is well established that in this model peak antibody production of specific classes occurs at specific time points (IgM = day 10, IgG = day 14, IgA = day 18[29]), any augmentation by PSP can be readily determined. It has been previously shown that for primary responses against NP, there exists an initial lag phase of 3–4 days T-cell priming [30]. Then the NP-specific B-cells will be recruited into splenic antibody responses, activated and grow exponentially. These B-cell clones then either develop into short-lived plasmablasts in extrafollicular foci, or migrate into follicles where they form germinal center long-lived plasma cells. Not only do the life-spans of the plasma cells from these two sources differ, the germinal center-derived plasma cells have been found to be of much higher affinities towards the specific immunogen [30]. This improved antigen-antibody blinding will have an important role for efficient B-cell effector functions, and thus clinically relevant implications. The aim of the study was to determine the antibody responses at various time points and assess the class of immunoglobulin they produced. Our study allows us to elucidate the mechanism of action of a clinically useful herbal medicine as an adjunct to cancer management. Acacia is a well- known immunopotentiator, where studies have shown that acacia gum can activate dendritic cells [31], confer innate and adaptive immunity (Strobel and Ferguson, 1982)[32] as well as exert anti-parasitic effects *in vivo* [33]. In addition, Strobel and others (1986) subsequently found that acacia gum can be used to induce oral tolerance depending on the model used [34]. In our study, acacia gum was used as an immunopotentiator to explore the combined effects with PSP to determine whether the T-cell dependent B-cells responses were enhanced compared to acacia gum alone

## Materials and Methods

### Animals and diet

5 week old C57BL/6 male mice were obtained from The Animal Resource Centre (Canning Vale, Australia) and maintained in a specific pathogen free facility with irradiated food and water ad libitum.

**Fig 2:**
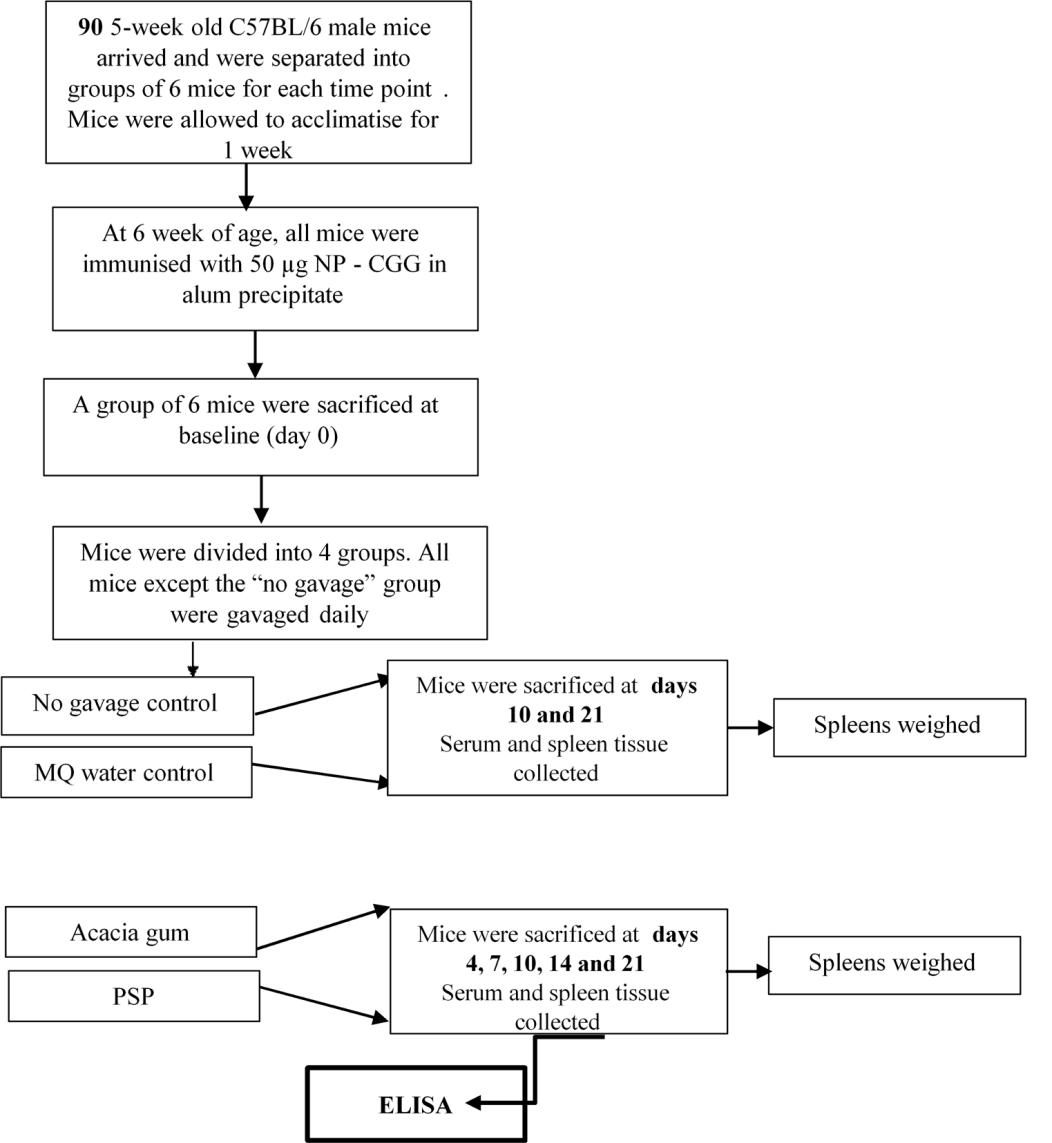
Schedule for acclimatization, immunization, PSP treatment and sacrifice of mice. 5 week old mice were allowed to acclimatize for one week, immunized with NP_25_-CGG at 6 weeks, groups of mice were then gavaged daily with PSP, ACACIA or MQ for up to 21 days and sacrificed at various time points (days 0, 4, 7, 10, 14 and 21).

All animal procedures were carried out in strict accordance with the recommendations in the Laboratory Animal Services Standard Operating Procedures and policies developed by the University of Sydney. The protocol was approved by the Animal Ethics Committee of the University of Sydney (Approval No: L24/2-2007/1/4484). Refer to flow diagram in Fig 2. All mice were monitored regularly and especially after gavaging and immunization for any adverse health issues and managed accordingly. Appropriate methods for euthanasia were carried out.

### Immunization and antigen

4-hydroxy-3-nitrophenylacetyl (NP) hapten conjugated to chicken γ globulin (CGG) (NP_25_-CGG; BioSearch Technologies, Novato, CA) was precipitated in alum [35]. 6 week old mice were immunised with 50 μg alum precipitate (in 200 μL PBS) injected i.p. at baseline (day 0) and then divided into 4 groups: NPCGG = no gavage control or untreated control, MQ = MQ water vehicle control, ACACIA = acacia gum, PSP = PSP treated

### PSP and serum collection

Polysaccharopeptide (PSP) derived from the Chinese *Cov-1* strain of *Coriolus versicolor* (Winsor Health Products Ltd, Hong Kong) was obtained and described previously [21]. PSP was suspended in 5% sterilized acacia gum in MQ water (Sigma, St. Louis, MO, USA) in a final concentration of 5mg/ml. 50 mg/kg (200 μl) of PSP was administered daily to the mice in the treatment group by oral gavage (PSP) and stopped after 21 days. Since acacia gum was the immunomodulatory potential baseline for the study, PSP was administered together with acacia gum. Any potential immunomodulatory effects of PSP would be expected to be greater than those of acacia gum. The remaining mice were controls and administered acacia gum (ACACIA), MQ water (MQ), or no gavage (NP-CGG). PSP was not administered to mice in the MQ group.

Groups of 6 mice were then sacrificed sequentially at baseline (day 0); 4, 7, 10, 14 and 21 days post immunization. PSP and ACACIA treated mice were sacrificed at days 4, 7, 10, 14 and 21; whereas MQ and NP-CGG treated mice were sacrificed at days 10 and 21. Serum was collected at all these time points.

### Conjugation of NP succinimide ester to protein carriers

Using the method by Lalor [36] the succinimide ester of NP (4-hydroxy-5-iodo-3-nitrophenyl) caproate-O-succinimide (NIP-OSu) (Biosearch Technologies, Novato, CA, USA) was conjugated to bovine serum albumin (BSA; Sigma) yielding an approximate substitution ratio of NP_2-_BSA and NP_18-_BSA respectively.

### Detection of NP-specific serum antibody by ELISA

NP-specific serum IgM, IgG, IgA and IgE were quantified by ELISA at various time points. To determine whether PSP was able to augment the predominantly early IgM antibody response to NP-CGG that peaks at day 10, we measured serum levels of mice at 0, 4, 7, 10, 14 and 21 days post immunization. Similarly to evaluate changes in the IgA responses to NP-CGG which peaks at day 18, we measured the serum titre levels of mice at 0, 10, 14 and 21 days post immunization. To examine whether PSP has the potential to augment any long term immunological responses that occur post 14 days of immunization, we measured high affinity and total IgG antibody responses to NP-CGG by direct ELISA using NP_2_-BSA and NP_18_-BSA respectively as the coating protein. Serum titres were determined at 0, 4, 7, 10, 14 and 21 days post immunization on NP_25_-CGG. To rule out any possible inflammatory or allergic responses that may have occurred in response to administering PSP for an extended period of time, we measured the IgE titre levels 7 days post immunization. Briefly 96-well plates were coated with either 100 μl of 5μg/ml NP_2_-BSA or NP_18_-BSA in 0.5 M carbonate buffer (pH 9.6) at 4°C overnight and blocked with 1% BSA in PBS. After four washes with PBS containing 0.05% Tween 20 (Santa Cruz Biotechnology, CA, USA), serially diluted serum samples were added to each well in duplicate and incubated for 1 h at 37°C in a humidified box. After washing, biotin-conjugated goat anti-mouse IgM, IgG, IgA (Caltag Laboratories) or IgE (Southern Biotech) dilutions ranging from 1:3000 to 1:10000 was added and incubated for 1 h at 37°C in a humidified box. SA-HRP activity was visualized using O-phenylenediamine (OPD; Sigma). After development the coloured reaction was stopped with 50 μl of 20% H_2_SO_4_ and optical densities were determined at 492 nm using a POLARstar plate reader (BMG Labtech, Australia). Positive control sera were included so that standard calibration curves for determination of relative antibody titres could be produced.

### Statistical analysis

Statistical analyses were performed using the non-parametric Kruskall-Wallis or *post-hoc* Mann-Whitney tests when appropriate. Statistical significance was achieved when p < 0.05 when compared to the control groups.

## Results

### Acacia and PSP have no effect on body and spleen weights

As PSP and Acacia are both known to be immunopotentiators, it is plausible that any enhanced immune responses caused by these agents in the NP-CGG model are likely to occur in the spleen and be reflected in changes in spleen weight. Body weight was also assessed to correspond with any changes in spleen weight and rule out any adverse effects of PSP and Acacia that may conversely be seen in a decrease in body weights. We did not find any significant variation in the body weights of mice before or during immunization (Table 1). Nor was there any significant variation in the body weights of mice in between groups or at different time points except at day 21, where NPCGG and MQ (P <0.05) were significantly increased compared to ACACIA (Table 2). There is a trend of increasing spleen weight seen from days 7–14 in the ACACIA group although this increase did not reach statistical significance. Overall there was no significant variation in spleen weights at death between groups (Table 3).

**Table 1:** Summary of mean body weights of mice at different time points.

Mice at various time points	Mice on arrival (5 weeks old, n = 90)	Mice after 1 week acclimatisation (6 weeks old, baseline: day 0) (n = 90)	Mice 1 week post immunization with NP-CGG (n = 90)	Mice 2 weeks after immunization with NP-CGG (n = 34)
Mean body weight (g) between groups (n = 6)	15.28 ± 0.23	19.16 ± 0.24	20.51 ± 0.62	22.35 ± 0.18

Table 1 summarises the mean ± SD body weights of C57BL/6 male mice upon arrival, 1 week after acclimatisation and at 1, 2 and 3 weeks post-immunization with NP-CGG. There were no significant variations in weight between groups and no decreases in weight indicating the absence of any post-immunization issues P > 0.05

**Table 2:** Summary of mean body weights of mice for each treatment group at death.

Days post immunization	NP-CGG(n = 6)	MQ (n = 6)	ACACIA (n = 6)	PSP (n = 6)
**0**	15.08 ± 0.85	ND	ND	ND
**4**	ND	ND	18.10 ± 2.11	19.07 ± 1.32
**7**	ND	ND	19.71 ± 2.03	21.10 ± 0.91
**10**	21.97 ± 1.49	20.95 ± 0.85	20.85 ± 0.93	21.82 ± 1.51
**14**	ND	ND	21.94 ± 0.84	22.47 ± 1.27
**21**	25.73 ± 0.65	22.52 ± 0.78	22.61 ± 0.79	23.38 ± 1.82

Table 2 summarises the mean body weights (g) ± SD for each treatment group of mice at death (n = 6) sacrificed on days 0, 4, 7, 10, 14 and 21. There were no significant differences in body weight between groups except at day 21 where NP-CGG and MQ mean body weights were significantly increased compared to ACACIA *P < 0.05, ND (not done)

**Table 3:** Summary of mean spleen weights of mice for each treatment group at death.

Days post immunization	NP-CGG (n = 6)	MQ (n = 6)	ACACIA (n = 6)	PSP (n = 6)
**0**	45.58 ± 3.95	ND	ND	ND
**4**	ND	ND	81.98 ± 5.78	86.33 ± 9.93
**7**	ND	ND	111.47 ± 53.78	81.07 ± 12.19
**10**	87.84 ± 10.09	96.41 ± 38.87	114.47 ± 32.79	103.71 ± 16.77
**14**	ND	ND	105.03 ± 55.55	84.68 ± 8.02
**21**	93.38 ± 17.20	90.73 ± 48.74	82.61 ± 12.21	79.14 ± 11.81

Table 3 summarises the mean spleen weights (mg) ± SD for each treatment group of mice at death sacrificed on days 0, 4, 7, 10, 14 and 21.There were no significant differences in spleen weight between groups P >0.05, ND (not done)

### PSP decreases NP-specific IgM titre levels at days 4 and 21

**Fig 3:**
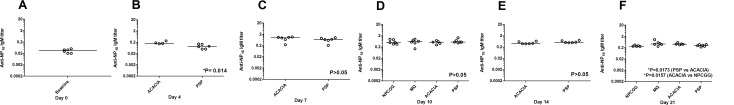
a-f. PSP significantly decreased NP-specific IgM responses at days 4 and 21 compared to ACACIA. C57BL/6 mice were immunised with NP_25_CGG and serum was collected on days 0, 4, 7, 10, 14 and 21. NP-specific IgM titers were measured from two-fold serially diluted (1/40-1/80) serum samples by ELISA. ACACIA significantly increased NP–specific IgM responses at day 21 compared to NP-CGG alone. Data are shown as the median from two independent experiments with 5–6 mice per group. *P < 0.05.

We assessed IgM titre levels since they are the first antibody to be produced by B-cells against an antigen in mice and humans alike and are especially potent in activating the complement system. IgM titre levels were assessed at each time point (days 4, 7, 10, 14 and 21) in order to determine whether PSP combined with Acacia could augment the known IgM titre level as per the NP-CGG model. In this model we would expect that the IgM titre level to peak at day 10. Any deviations such as a shift in this peak or earlier or later changes will be reflected in this maximum occurring at different time points. Interestingly we found a significant decrease in the serum NP specific IgM titre levels in the mice treated with PSP at days 4 and 21 (Fig 3B and 3F) compared to those treated with the ACACIA (P < 0.05). In contrast, we found a significant increase in the ACACIA group compared to the NP-CGG control group (P <0.05) at day 21(Fig 3F). The MQ group showed a slight increase in serum NP specific IgM titre levels at day 10 which is more apparent on day 21 (Fig 3D and 3F), although they were not significantly different from any of the other groups. There were no significant differences in the NP specific IgM titre levels at 7, 10 or 14 days post immunization in the PSP or ACACIA groups nor in the MQ or NP-CGG control groups (P >0.05) (Fig 3C–3E).

### PSP increases NP-specific IgG titre levels at day 4

**Fig 4:**
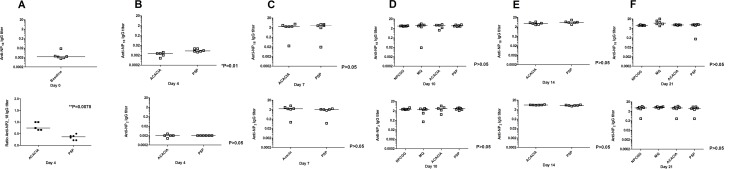
a-f. PSP significantly increase NP-specific IgG responses compared to the vehicle control. Expression of the total high affinity and low affinity NP-specific IgG titers were measured from two-fold serially diluted serum samples (from 1/40-1/80 for day 4 to 1/8000-1/10000 for day 10 onwards) using NP_2_-per BSA (NP_2_) and NP_18_-per BSA (NP_18_) respectively by ELISA. The low affinity NP-specific IgG antibody responses to ACACIA were significant at day 4 but at no other time point (day 7, 10, 14 or 21) compared to PSP. The ratio between the expression of total high affinity and low affinity NP-specific IgG responses was significant for ACACIA at day 4 compared to PSP. NP_2_ baseline very low (data not shown). Data are shown as the median from two independent experiments with 5–6 mice per group. *P < 0.05.

We assessed IgG levels as since they follow the production of IgM and IgA though more potent in longer term secondary immune responses, IgG titre levels were assessed at each time point (days 4, 7, 10, 14 and 21) in order to determine whether PSP combined with Acacia could augment the known IgG titre level as per the NP-CGG model. In this model we would expect that the IgG titre level to peak at day 14. Any deviations such as a shift in this peak or earlier or later changes will be reflected in this maximum occurring at different time points. We found a significant increase in the low affinity NP-specific IgG serum titre levels in mice treated with PSP at day 4 compared to ACACIA (P < 0.05) as seen in Fig 4. This increase in titre level at day 4 occurs much earlier than expected. The ratio between the expression of the total high affinity to the low affinity NP-specific IgG antibody response was significant for ACACIA at day 4 (P = 0.008) compared to PSP (Fig 4). The MQ group showed a slight increase in NP specific IgG serum titre levels at day 10 which is more apparent on day 21 (Fig 4D and 4F) for both the total high affinity and low affinity responses although they were not significantly different from any of the other groups.

Overall, we did not find any significant differences in the expression of the total high affinity or low affinity NP-specific IgG serum levels to NP-CGG at any of the other time points or groups (P >0.05).

### PSP does not alter NP-specific IgA titre levels

**Fig 5:**
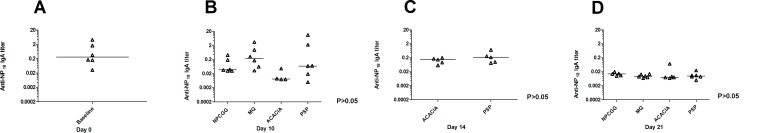
a-d. Absence of any significant NP- specific IgA responses to PSP or ACACIA. After NP-CGG immunization no changes in IgA antibody responses were observed at any time point (day 0, 10, 14 or 21) to PSP or ACACIA. NP- specific IgA titers were measured from two-fold serially diluted (1/50-1/100) serum samples by ELISA. Data are shown as the median from two independent experiments with 5–6 mice per group.

We assessed IgA titre levels since they are the first level of defense acting as a neutralizing antibody against antigens invading through the mucosa. IgA titre levels were assessed at each time point (days 4, 7, 10, 14 and 21) in order to determine whether PSP combined with Acacia could augment the known IgA titre level as per the NP-CGG model. In this model we would expect that the IgA titre level to peak at day 18. Any deviations such as a shift in this peak or earlier or later changes will be reflected in this maximum occurring at different time points. As such, we did not find any significant differences in the NP-specific IgA serum levels at any of the time points investigated nor in any of groups (P >0.05) (Fig 5).

### PSP does not alter NP-specific IgE titre levels

**Fig 6:**
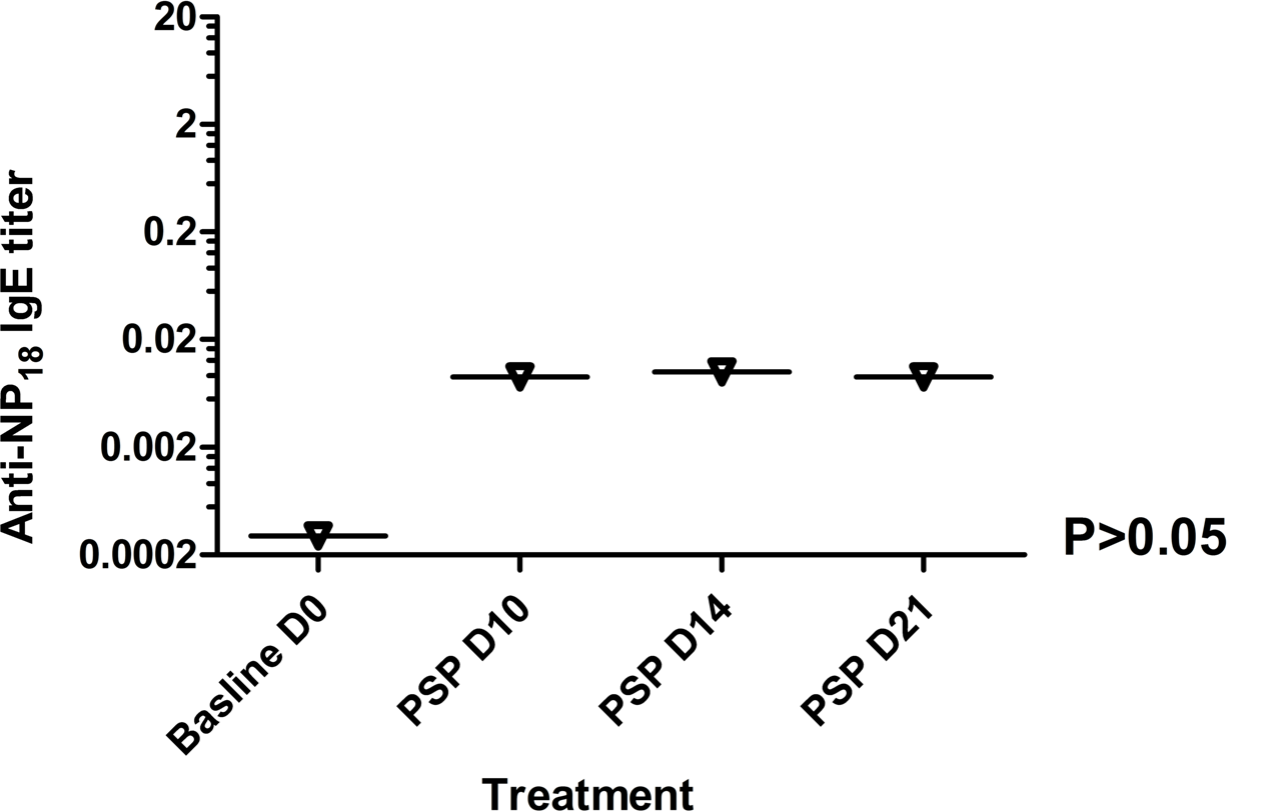
Absence of any significant NP-specific IgE responses to PSP. After NP-CGG immunization no changes in IgE antibody responses were observed at any time point (day 4, 7, 10, 14 or 21) to PSP NP- specific IgE titers were measured from two-fold serially diluted (1/5-1/20) serum samples by ELISA. Data are shown as the median from two independent experiments with 5–6 mice per group.

We assessed IgE titre levels to rule out whether PSP is capable of inducing allergenic effects in this model since antigen binding may trigger the mast cell to release chemical mediators such as histamine that induce anti-parasite immunity and allergy. We did not find any significant changes in IgE levels at days 10, 14 or 21 in the PSP treated group compared to baseline (NP-CGG group at day 0 post immunization) (P >0.05) as seen in Fig 6. It is unlikely that PSP exerts allergic responses in this model.

## Discussion

Cancer immunosurveillance occurs when the immune system’s checking processes are impaired, thereby resulting in an inability to correctly identify, effectively target and destroy cancer cells which can lead to tumor growth and metastasis. More recently, “cancer immunoediting” has been used to describe this dynamic process of elimination, equilibrium and escape [37]. While immunotherapy is a sound targeted approach to address and augment each of these processes, the use of herbal medicines offers great promise. Polysaccharopeptides such as PSK and PSP derived from the mushroom *Coriolus versicolor* have been well studied and purported to enhance the immune system with broad ranging anti-cancer activities, conferring a survival advantage in cancer patients [38]. PSK (otherwise known as Krestin) has been used in Japan as an adjuvant to conventional anti-cancer treatments since 1977 [39].

Similarly, PSP has been used as an immune enhancer in China and has been subject to 30 years of research; however despite extensive *in vitro*, *in vivo* and clinical studies, the specific immunological mechanism of action has still not been clearly identified. We are the first to report the application of a well-established *in vivo* model NP-CGG to herbal medicines with immunotherapeutic potential. We report that PSP does not exert specific immunological effects through T-cell dependent B-cell responses. This can be seen in the absence of any significant changes in the various anti NP-immunoglobulins tested including IgM, IgG, IgA or IgE (Figs 3–6). Although we observed a statistically significant decrease in the serum specific IgM titre level in mice treated PSP on days 4 and 21 (Fig 3B and 3F) compared to those treated with ACACIA (P < 0.05), these results though small are indicative of a trend. Additionally, we observed a significant increase in the total IgG serum titre level in mice treated with PSP on day 4 compared to ACACIA (P < 0.05) as seen in Fig 4. In terms of the known mechanics of the NP-CGG model, IgG responses are likely to occur at day 14 onwards [30], these early changes observed are small but lower than expected. While PSP did not significantly alter the IgE titre levels in mice treated with PSP (Fig 6), it is unlikely that PSP contributes to allergic responses. In this study we have demonstrated a consistent increasing trend across all Ig classes, except IgE, to NP-CGG by PSP. This is consistent with isotype switching IgM to IgG. IgA responses drop off over time as expected due to tolerance caused by consistent mucosal stimulation of gavaging. PSP therefore produces small effects on the T-cell dependent B-cell responses *in vivo*. There is a trend of increasing spleen weight seen from days 7–14 in the ACACIA group weight with a corresponding decrease in body weight, although this increase did not reach statistical significance. Additionally, we did not observe any significant alterations in body or spleen weights in any of the groups measured due to the addition of PSP (Tables 2 and 3) or adverse reactions associated with immunization. Increased body weights within groups were as expected.

Interestingly, the effects of acacia gum on T-cell dependent responses are of significance. In contrast to PSP, we found a significant increase in the IgM titre level in the ACACIA group compared to the NP-CGG group (P <0.05) at day 21 (Fig 3F), which is later than expected as IgM peaks at day 10. Interestingly, the ratio between the expression of the total high affinity and low affinity -NP-specific IgG antibody response was significant for ACACIA at day 4 (P = 0.008) compared to PSP (Fig 4), also occurring earlier than expected. There was a small but non-significant increase in the serum NP specific IgM and IgG titre levels for MQ, especially at day 21, that may due to the larger variation of individual responses observed within that particular group (P >0.05). In addition, we did not find any significant differences in the expression of the total high affinity or low affinity IgG serum levels to NP-CGG at any of the other time points or groups (P >0.05). While these results suggest that ACACIA might influence T-cell dependent B-cell responses, the fold differences are small and need to be confirmed. Studies have shown that acacia gum can activate dendritic cells [31], confer innate and adaptive immunity as well as exert anti-parasitic effects *in vivo*. Unsurprisingly, acacia gum is known to be an immunopotentiator due to the presence of 1–3% arabinogalactan protein integral to its structure, while allergenicity of acacia gum does not feature in the natural route via the gut [34].Furthermore, a 90 day toxicity study where rats were fed a dietary level of 5% acacia gum reported no adverse effects or histopathological alterations in the lymphoid tissues [40]. As such, a few studies have reported the use of acacia gum as a vehicle control when investigating potential immunological effects of herbal medicines, although without the augmented responses by the control [41,42]. Acacia gum’s use as an immunopotentiator or adjuvant may be more appropriate than as a control in such immunological studies. Our study demonstrates a combined adjuvant response of acacia gum when combined with the immunomodulating herbal medicine PSP. Although our results indicate a small trend, further studies are needed to confirm their combined effects using other animal models.

The role of adjuvants in their ability to stimulate the immune response to enhance vaccination therapies is well established. Noteworthy is the Kettering study that demonstrated the use of an adjuvant QS-21 in Phase I trial administered with a constant dose of the melanoma ganglioside GM2 covalently attached to KLH [43]. Antibodies were induced against both the melanoma ganglioside and the protein KLH and were well tolerated in advanced melanoma patients. Since then many different adjuvants are entering the clinic including those that are derived from polysaccharides such as Advax. However PSP’s potential role as a vaccine adjuvant cannot be dismissed. A recent study looking at the effects of PSK found that it was not only able to activate dendritic cells *in vitro* and *in vivo* but acted as an adjuvant to OVAp323 vaccination inducing proliferation of antigen specific T-cells by augmenting the number of antigen specific T-cells in the spleen and draining lymph nodes as well as enhancing cytokine responses [44].

Our results support our previous i*n vitro* studies reporting that PSP does not directly alter T-cell subsets. We previously reported that PSP also does not alter NK cells or B-cells but instead upregulates the percentage of monocytes [21]. Specifically, we did not find any significant differences in the CD4/8 ratio after PSP treatment *in vitro* [21] which is in agreement with clinical findings [22,23]. Based on the existing research, it is likely that PSP exerts it broad effects in a non-specific manner. To confirm whether PSP and acacia gum exerts non-specific adjuvant effects, further investigations should be conducted to examine whether this combination further enhances T-cell independent B-cell responses. This can be done using the same model but by investigating the effect of this combination on different proteins conjugated to NP, particularly Ficoll or other related responses such as hen egg lysozyme (HEL) and pigeon cytochrome C (PCC). Observing the corresponding changes in the germinal centre of spleens as well as immunoglobulins in sera would be ideal for this combination compared to the individual agents. Other recent applications of the NP-CGG model include assessing the potential immunosuppressive activities of certain drugs including quinoline compounds. While quinoline-3-carbaoxamide was found to modulate primary T-cell dependent B-cell responses, functional immunity was not inhibited [45]. There is therefore great potential and application for using the NP-CGG model in the area of investigating novel therapeutic agents with immunomodulatory potential, such as herbal medicines, including medicinal mushrooms combined with agents with known adjuvant properties such as acacia gum to enhance immune responses.

Currently the NP-CGG models have been used exclusively to study B-cell repertoire and germinal centre changes in response to immunization with various agents and adjuvants. We describe for the first time the application of this model to the investigation of the immune enhancing effects of combined glycoprotein-based herbal medicines, most importantly to differentiate these effects from the non-specific stimulatory T-cell and B-cell effects.

## References

[1] Hanahan D, Weinberg RA (2011) Hallmarks of cancer: the next generation. Cell 144: 646–674. 10.1016/j.cell.2011.02.013 21376230

[2] Dunn GP, Old LJ, Schreiber RD (2004) The immunobiology of cancer immunosurveillance and immunoediting. Immunity 21: 137–148. 15308095 10.1016/j.immuni.2004.07.017

[3] Mittal D, Gubin MM, Schreiber RD, Smyth MJ (2014) New insights into cancer immunoediting and its three component phases—elimination, equilibrium and escape. Curr Opin Immunol 27: 16–25. 10.1016/j.coi.2014.01.004 24531241 PMC4388310

[4] Nesher L, Rolston KV (2014) The current spectrum of infection in cancer patients with chemotherapy related neutropenia. Infection 42: 5–13. 10.1007/s15010-013-0525-9 23975584

[5] Anguille S, Smits EL, Lion E, van Tendeloo VF, Berneman ZN (2014) Clinical use of dendritic cells for cancer therapy. Lancet Oncol 15: e257–e267. 10.1016/S1470-2045(13)70585-0 24872109

[6] Rosenberg SA, Yang JC, Sherry RM, Kammula US, Hughes MS, Phan GQ, et al (2011) Durable complete responses in heavily pretreated patients with metastatic melanoma using T-cell transfer immunotherapy. Clin Cancer Res 17: 4550–4557. 10.1158/1078-0432.CCR-11-0116 21498393 PMC3131487

[7] Lasek W, Zagozdzon R, Jakobisiak M (2014) Interleukin 12: still a promising candidate for tumor immunotherapy? Cancer Immunol Immunother 63: 419–435. 10.1007/s00262-014-1523-1 24514955 PMC3994286

[8] Hanzly M, Aboumohamed A, Yarlagadda N, Creighton T, Digiorgio L, Fredrick A, et al (2014) High-dose interleukin-2 therapy for metastatic renal cell carcinoma: a contemporary experience. Urology 83: 1129–1134. 10.1016/j.urology.2014.02.005 24767525

[9] Ribatti D (2014) From the discovery of monoclonal antibodies to their therapeutic application: An historical reappraisal. Immunol Lett 161: 96–99. 10.1016/j.imlet.2014.05.010 24877873

[10] Acharya UH, Jeter JM (2013) Use of ipilimumab in the treatment of melanoma. Clin Pharmacol 5: 21–27. 10.2147/CPAA.S45884 PMC3558314 24113540

[11] Mamalis A, Garcha M, Jagdeo J (2014) Targeting the PD-1 pathway: a promising future for the treatment of melanoma. Arch Dermatol Res 306: 511–519. 10.1007/s00403-014-1457-7 24615548 PMC4110159

[12] Sze DM, Brown R, Yang S, Ho PJ, Gibson J, Joshua D (2006) The use of thalidomide in myeloma therapy as an effective anticancer drug. Curr Cancer Drug Targets 6: 325–331. 16848723 10.2174/156800906777441762

[13] Brown RD, Spencer A, Ho PJ, Kennedy N, Kabani K, Yang S, et al (2009) Prognostically significant cytotoxic T cell clones are stimulated after thalidomide therapy in patients with multiple myeloma. Leuk Lymphoma 50: 1860–1864. 10.3109/10428190903216804 19883313

[14] Li J, Sze DM, Brown RD, Cowley MJ, Kaplan W, Mo SL, et al (2010) Clonal expansions of cytotoxic T cells exist in the blood of patients with Waldenstrom macroglobulinemia but exhibit anergic properties and are eliminated by nucleoside analogue therapy. Blood 115: 3580–3588. 10.1182/blood-2009-10-246991 20190191

[15] Vannucci L, Krizan J, Sima P, Stakheev D, Caja F, Rajsiglova L, et al (2013) Immunostimulatory properties and antitumor activities of glucans (Review). Int J Oncol 43: 357–364. 10.3892/ijo.2013.1974 23739801 PMC3775562

[16] Lee KH, Morris-Natschke SL, Yang X, Huang R, Zhou T, Wu SF, et al (2012) Recent progress of research on medicinal mushrooms, foods, and other herbal products used in traditional Chinese medicine. J Tradit Complement Med 2: 84–95. 24716120 PMC3942920

[17] Cui J, Chisti Y (2003) Polysaccharopeptides of Coriolus versicolor: physiological activity, uses, and production. Biotechnol Adv 21: 109–122. 14499133 10.1016/s0734-9750(03)00002-8

[18] Lee C-L, Yang X, Wan JM-F (2006) The culture duration affects the immunomodulatory and anticancer effect of polysaccharopeptide derived from Coriolus versicolor. Enzyme and Microbial Technology 38: 14–21.

[19] Ooi VE, Liu F (2000) Immunomodulation and anti-cancer activity of polysaccharide-protein complexes. Curr Med Chem 7: 715–729. 10702635 10.2174/0929867003374705

[20] QY Y (1999) History, present status and perspectives of the study of Yun Zhi polysaccharopeptide In: QY Y, editor. Advanced Research in PSP, 1999. Hong Kong The Hong Kong Association for Health Care Ltd pp. 5–15.

[21] Sekhon BK, Sze DM, Chan WK, Fan K, Li GQ, Moore DE, et al (2013) PSP activates monocytes in resting human peripheral blood mononuclear cells: immunomodulatory implications for cancer treatment. Food Chem 138: 2201–2209. 10.1016/j.foodchem.2012.11.009 23497877

[22] Liao ML ZJ (1993) The II stage clinical tests of PSP in the treatment of lung cancer. In: Yang QY KC, editor. Proceedings of PSP International Symposium. Shanghai: Fudan University Press. pp. 243–256.

[23] Zhang LY, Zhong Y., & Zhou J. (1999) The observation of PSP decrease 60 cases chemotherapeutical stomach cancer’s side effect In: QY Y, editor. Advanced Research in PSP, 1999. Hong Kong: The Hong Kong Association for Health Care Ltd pp. 328–329.

[24] Ragupathi G, Yeung KS, Leung PC, Lee M, Lau CB, Vickers A, et al (2008) Evaluation of widely consumed botanicals as immunological adjuvants. Vaccine 26: 4860–4865. 10.1016/j.vaccine.2008.06.098 18640165 PMC2565601

[25] Hong F, Xiao W, Ragupathi G, Lau CB, Leung PC, Yeung KS, et al (2011) The known immunologically active components of Astragalus account for only a small proportion of the immunological adjuvant activity when combined with conjugate vaccines. Planta Med 77: 817–824. 10.1055/s-0030-1250574 21128203 PMC3711077

[26] Zhu Z, Cuss SM, Singh V, Gurusamy D, Shoe JL, Leighty R, et al (2015) CD4+ T Cell Help Selectively Enhances High-Avidity Tumor Antigen-Specific CD8+ T Cells. J Immunol 195: 3482–3489. 10.4049/jimmunol.1401571 26320256 PMC7687044

[27] Naslund TI, Gehrmann U, Qazi KR, Karlsson MC, Gabrielsson S (2013) Dendritic cell-derived exosomes need to activate both T and B cells to induce antitumor immunity. J Immunol 190: 2712–2719. 10.4049/jimmunol.1203082 23418627

[28] Bertrand F, Rochotte J, Colacios C, Montfort A, Tilkin-Mariame AF, Touriol C, et al (2015) Blocking Tumor Necrosis Factor alpha Enhances CD8 T-cell-Dependent Immunity in Experimental Melanoma. Cancer Res 75: 2619–2628. 10.1158/0008-5472.CAN-14-2524 25977337

[29] Toellner KM, Gulbranson-Judge A, Taylor DR, Sze DM, MacLennan IC (1996) Immunoglobulin switch transcript production in vivo related to the site and time of antigen-specific B cell activation. J Exp Med 183: 2303–2312. 8642339 10.1084/jem.183.5.2303 PMC2192570

[30] Sze DM, Toellner KM, Garcia de Vinuesa C, Taylor DR, MacLennan IC (2000) Intrinsic constraint on plasmablast growth and extrinsic limits of plasma cell survival. J Exp Med 192: 813–821. 10993912 10.1084/jem.192.6.813 PMC2193289

[31] Xuan NT, Shumilina E, Nasir O, Bobbala D, Gotz F, Lang F (2010) Stimulation of mouse dendritic cells by Gum Arabic. Cell Physiol Biochem 25: 641–648. 10.1159/000315083 20511709

[32] Strobel S, Ferguson A, Anderson DM (1982) Immunogenicity of foods and food additives—in vivo testing of gums arabic, karaya and tragacanth. Toxicol Lett 14: 247–252. 7167986 10.1016/0378-4274(82)90059-5

[33] Ballal A, Bobbala D, Qadri SM, Foller M, Kempe D, Nasir O, et al (2011) Anti-malarial effect of gum arabic. Malar J 10: 139 10.1186/1475-2875-10-139 21599958 PMC3116497

[34] Strobel S, Ferguson A (1986) Induction of oral tolerance, in mice, to gum arabic. Food Addit Contam 3: 43–46. 3956792 10.1080/02652038609373563

[35] Jacob J, Kassir R, Kelsoe G (1991) In situ studies of the primary immune response to (4-hydroxy-3-nitrophenyl)acetyl. I. The architecture and dynamics of responding cell populations. J Exp Med 173: 1165–1175. 1902502 10.1084/jem.173.5.1165 PMC2118845

[36] Lalor PA, Nossal GJ, Sanderson RD, McHeyzer-Williams MG (1992) Functional and molecular characterization of single, (4-hydroxy-3-nitrophenyl)acetyl (NP)-specific, IgG1+ B cells from antibody-secreting and memory B cell pathways in the C57BL/6 immune response to NP. Eur J Immunol 22: 3001–3011. 1425924 10.1002/eji.1830221136

[37] Dunn GP, Old LJ, Schreiber RD (2004) The three Es of cancer immunoediting. Annu Rev Immunol 22: 329–360. 15032581 10.1146/annurev.immunol.22.012703.104803

[38] Eliza WL, Fai CK, Chung LP (2012) Efficacy of Yun Zhi (Coriolus versicolor) on survival in cancer patients: systematic review and meta-analysis. Recent Pat Inflamm Allergy Drug Discov 6: 78–87. 22185453 10.2174/187221312798889310

[39] Yang QY JS, Li XY, Zhou JX, Chen RT, Xu LZ (1992a) Antitumor and immunomodulating activities of the polysaccharide-peptide (PSP) of *Coriolus versicolor*. J Immunol Immunopharmacol 12: 29–34.

[40] Doi Y, Ichihara T, Hagiwara A, Imai N, Tamano S, Orikoshi H, et al (2006) A ninety-day oral toxicity study of a new type of processed gum arabic, from Acacia tree (Acacia senegal) exudates, in F344 rats. Food Chem Toxicol 44: 560–566. 16256256 10.1016/j.fct.2005.09.002

[41] Ouyang HQ, Liu LM, Chen Z, Luo JM, Yu EX (2010) Effects of Qingyi Huaji decoction on serum levels of interleukin-6, interleukin-8 and tumor necrosis factor-alpha in nude mice bearing pancreatic tumors. Zhong Xi Yi Jie He Xue Bao 8: 655–661. 20619142 10.3736/jcim20100709

[42] Tanaka T, Sugiura H, Inaba R, Nishikawa A, Murakami A, Koshimizu K, et al (1999) Immunomodulatory action of citrus auraptene on macrophage functions and cytokine production of lymphocytes in female BALB/c mice. Carcinogenesis 20: 1471–1476. 10426794 10.1093/carcin/20.8.1471

[43] Livingston PO, Adluri S, Helling F, Yao TJ, Kensil CR, Newman MJ, et al (1994) Phase 1 trial of immunological adjuvant QS-21 with a GM2 ganglioside-keyhole limpet haemocyanin conjugate vaccine in patients with malignant melanoma. Vaccine 12: 1275–1280. 7856291 10.1016/s0264-410x(94)80052-2

[44] Engel AL, Sun GC, Gad E, Rastetter LR, Strobe K, Yang Y, et al (2013) Protein-bound polysaccharide activates dendritic cells and enhances OVA-specific T cell response as vaccine adjuvant. Immunobiology 218: 1468–1476. 10.1016/j.imbio.2013.05.001 23735481 PMC3783519

[45] Kallberg E, Ivars F, Leanderson T (2014) Quinoline-3-carboxamides modulate primary T cell-dependent B cell responses but do not inhibit functional immunity. Scand J Immunol 79: 237–243. 10.1111/sji.12152 24383944

